# *Piper sarmentosum* Promotes Endothelial Nitric Oxide Production by Reducing Asymmetric Dimethylarginine in Tumor Necrosis Factor-α-Induced Human Umbilical Vein Endothelial Cells

**DOI:** 10.3389/fphar.2019.01033

**Published:** 2019-09-17

**Authors:** Uma Mahgesswary Sundar, Azizah Ugusman, Hui Kien Chua, Jalifah Latip, Amilia Aminuddin

**Affiliations:** ^1^Department of Physiology, Faculty of Medicine, Universiti Kebangsaan Malaysia Medical Centre, Kuala Lumpur, Malaysia; ^2^Department of Pharmaceutical Chemistry, School of Chemical Sciences & Food Technology, Faculty of Science and Technology, Universiti Kebangsaan Malaysia, Bangi, Malaysia

**Keywords:** Piper sarmentosum, asymmetric dimethylarginine, dimethylarginine dimethylaminohydrolase, human umbilical vein endothelial cells, tumor necrosis factor-α, endothelial dysfunction

## Abstract

Asymmetric dimethylarginine (ADMA) is an endogenous inhibitor of endothelial nitric oxide synthase (eNOS). ADMA is degraded by dimethylarginine dimethylaminohydrolase (DDAH). Elevated levels of ADMA lead to reduction in nitric oxide (NO) production, which is linked to endothelial dysfunction and atherosclerosis. *Piper sarmentosum* is an herb that has shown stimulation on endothelial NO production by increasing both expression and activity of eNOS. Thus, this study determined whether the positive effect of *P. sarmentosum* on NO production is related to its modulation on the DDAH–ADMA pathway in cultured human umbilical vein endothelial cells (HUVEC) exposed to tumor necrosis factor-α (TNF-α). HUVEC were divided into four groups: control, treatment with 250 µg/ml of aqueous extract of *P. sarmentosum* leaves (AEPS), treatment with 30 ng/ml of TNF-α, and concomitant treatment with AEPS and TNF-α for 24 h. After treatments, HUVEC were collected to measure *DDAH1* messenger RNA (mRNA) expression using quantitative real-time polymerase chain reaction. DDAH1 protein level was measured using enzyme-linked immunosorbent assay (ELISA), and DDAH enzyme activity was measured using colorimetric assay. ADMA concentration was measured using ELISA, and NO level was measured using Griess assay. Compared to control, TNF-α-treated HUVEC showed reduction in *DDAH1* mRNA expression (*P* < 0.05), DDAH1 protein level (*P* < 0.01), and DDAH activity (*P* < 0.05). Treatment with AEPS successfully increased *DDAH1* mRNA expression (*P* < 0.05), DDAH1 protein level (*P* < 0.01), and DDAH activity (*P* < 0.05) in TNF-α-treated HUVEC. Treatment with TNF-α caused an increase in ADMA level (*P* < 0.01) and a decrease in endothelial NO production (*P* < 0.001). Whereas treatment with AEPS was able to reduce ADMA level (*P* < 0.01) and restore NO (*P* < 0.001) in TNF-α-treated HUVEC. The results suggested that AEPS promotes endothelial NO production by stimulating DDAH activity and thus reducing ADMA level in TNF-α-treated HUVEC.

## Introduction

Atherosclerotic cardiovascular disease is the leading cause of mortality worldwide (Benjamin et al., 2017). The earliest stage of atherosclerosis development is endothelial dysfunction that is associated with other cardiovascular risk factors such as hypertension, hypercholesterolemia, hyperhomocysteinemia, diabetes mellitus, obesity, and systemic inflammation, contributing to the pathogenesis of cardiovascular diseases (Su, 2015). Endothelial dysfunction is defined as the loss of the homeostatic mechanisms that operate in healthy endothelial cells. It is characterized by decreased bioavailability of nitric oxide (NO), which promotes vascular dysfunction and atherosclerosis progression (Su, 2015).

Endothelium-derived NO is synthesized by endothelial nitric oxide synthase (eNOS). NO acts as an endogenous anti-atherosclerotic molecule by inducing vasodilation (Sukhovershin et al., 2015) and inhibiting monocyte adhesion, smooth muscle cell proliferation, and platelet aggregation (Ishizaka et al., 2007). Decrease of NO leads to endothelial dysfunction and subsequently promotes atherosclerosis. Asymmetric dimethylarginine (ADMA) is a competitive inhibitor of eNOS, which reduces NO production. ADMA level was increased in individuals with hypercholesterolemia, hypertension, diabetes mellitus, and hyperhomocysteinemia, leading to increased risk of atherosclerosis (Jawalekar et al., 2013).

A small part of ADMA is excreted *via* the kidneys, while most ADMA is degraded by dimethylarginine dimethylaminohydrolase (DDAH) enzyme to dimethylamine and l-citrulline (Liu et al., 2016). Reduction in DDAH activity leads to an increase in ADMA, which in turn reduces eNOS activity and NO production (Czarnecka et al., 2017). Tumor necrosis factor-α (TNF-α) is a pro-inflammatory cytokine that reduces the expression and activity of eNOS. TNF-α also reduces DDAH activity and consequently increases ADMA level (Vairappan, 2015). There are two isoforms of DDAH, with DDAH1 predominantly found in the kidneys and brain while DDAH2 is present mainly in the kidneys and heart (Bulau et al., 2007). Several studies have identified the role of DDAH1 in ADMA degradation and NO synthesis while the physiological function of DDAH2 is still undetermined (Liu et al., 2016). Enzyme kinetics of these isoforms demonstrated a *V*_max_ value of 356 and 4.8 nmol/mg/min for ADMA of DDAH1 and DDAH2, respectively. Thus, the apparent rate of ADMA degradation for DDAH1 is 70 times higher than that of DDAH2 (Pope et al., 2009). Based on these enzyme kinetics studies, DDAH1 is recognized as the principal ADMA metabolizing pathway in the endothelium. In addition, overexpression of *DDAH1* was reported to reduce ADMA level in mice (Zhang et al., 2011). Hence, this study was focused mainly on *DDAH1* expression.

*Piper sarmentosum* is an herbaceous plant that is widely used in Chinese traditional medicine to treat fever, cough, pleurisy, toothache, and dyspepsia. The vernacular names of *P. sarmentosum* vary among different countries such as *daun kaduk* in Malaysia, *cha plu* in Thailand, and *qing ju* in China. The plant easily grows in tropical and subtropical regions, especially in shady and moist areas (Chaveerach et al., 2008). Aqueous extract of *P. sarmentosum* (AEPS) leaves is rich in flavonoids and possesses numerous pharmacological properties such as anti-inflammatory, antioxidant, antibacterial, and anti-osteoporosis activities (Chan and Wong, 2014). AEPS leaves also reduced the formation of atherosclerosis in hypercholesterolemic rabbits (Adel et al., 2010). The extract was able to reduce blood pressure and increase serum nitric oxide in spontaneously hypertensive rats (Zainudin et al., 2015). Subacute toxicity study in rats showed that AEPS leaves was safe for consumption (Zainudin et al., 2013). In addition, AEPS leaves promoted the production of NO in human umbilical vein endothelial cells (HUVEC) by increasing both expression and activity of eNOS (Ugusman et al., 2010). Therefore, this study was conducted to determine whether the positive effect of *P. sarmentosum* on NO production is related to its modulation on the DDAH–ADMA pathway in HUVEC treated with TNF-α. We hypothesized that AEPS stimulated endothelial NO generation by increasing DDAH and decreasing ADMA, hence protecting against endothelial dysfunction and atherosclerosis.

## Materials and Method

### Preparation and Chemical Analysis of Aqueous Extract of P. *sarmentosum*

**Figure 1:**
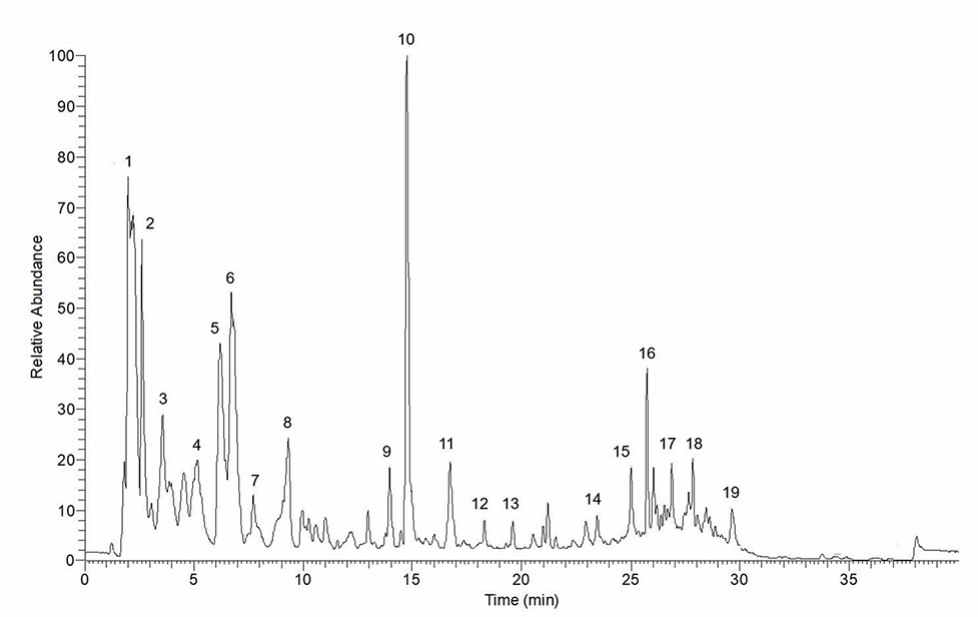
Total ion chromatogram of aqueous extract of *P. sarmentosum* leaves (AEPS). Peaks are identified with numbers according to the elution order (refer to **Table 1**).

Fresh leaves of *P. sarmentosum* were purchased in one batch from Herbagus Sdn. Bhd., Penang, Malaysia, and only this batch was used throughout the study. The leaves were identified by plant taxonomists in Herbarium, Universiti Kebangsaan Malaysia (UKM) (specimen voucher number: UKMB40240). AEPS was prepared according to the previous method (Ugusman et al., 2010). Fresh *P. sarmentosum* leaves were rinsed with water, dried under sunlight, and blended into powder. Then, 100 g of the powder were immersed in 900 ml of Milli-Q water (1:10, w/v) in a reflux extractor machine. The extract was boiled at 80°C for 3 h, and the resulting solution was filtered and frozen at −80°C. Finally, the extract was freeze-dried and stored at 4°C. Liquid chromatography (LC)–mass spectrometry (MS) Orbitrap full-scan analysis was conducted to identify the compounds present in AEPS. LC–ultraviolet (UV) analysis was performed using the Accela^™^ ultrahigh-performance LC (UHPLC) system (Thermo Scientific, San Jose, USA) equipped a with quarternary pump, a build degasser, a photodiode array (PDA) detector, and an autosampler. The column used for the chromatographic separation was a Luna Kinetex Reversed Phase C18 column [2.6-µm particle size, 2.1- mm inner diameter (ID) × 150 mm]. The conditions consisted of a gradient elution using acetonitrile as mobile phase A and 0.1% formic acid as phase B at a flow rate of 200 µl/min over 40 min. The following gradient was applied: 0–20 min, 5% A; 20–30 min, 35% A; 30–35 min, 100% A; 35–35.01 min, 5% A, returning to 5% A in 5 min, with 10 µl of sample injected. The compounds eluted and separated were further analyzed with a linear trap quadrupole) LTQ Orbitrap mass spectrometer (Thermo Scientific, San Jose, USA) operating in a negative ion mode. The operating parameters were as follows: source accelerating voltage, 4.0 kV; capillary temperature, 350°C; sheath gas flow, 40 arb; auxiliary gas, 20 arb. Mass spectra were acquired with a scan range of 300–1,000 *m*/*z*. All data were processed with the Xcalibur^™^ 2.1 software (Thermo Scientific, San Jose, USA). LC–MS Orbitrap full-scan analysis identified a total of 19 compounds in AEPS (**Figure 1**). The compounds were characterized using retention times (R*_t_*) and mass spectra, as provided by electrospray ionization (ESI)–MS (summarized in **Table 1**).

**Table 1:** Retention time (R*_t_*) and mass spectra details for compounds identified in the negative mode of liquid chromatography (LC)–mass spectrometry (MS) Orbitrap full-scan analysis.

Peak	R*_t_* (min)	m/z	Molecular weight	Molecular formula	Proposed compound
1	1.98	195.0503 (M-Na)	218.252	C_13_H_14_O_3_	2,2-Dimethyl-2*H*-1-benzopyran-6-carboxylic acid; methyl ester
2	2.61	191.01933 (M-H)	192.214	C_11_H_12_O_3_	1-Allyl-2-methoxy-4,5-methylenedioxybenzene/sarisan/asaricin
3	3.56	203.0820 (M-H)	204.35	C_15_H_24_	Sesquisabinene
4	5.16	315.0530 (M-H)	316.5	C_21_H_32_O_2_	1-(3,4-Methylenedioxyphenyl)-1*E*-tetradecene
5	6.18	341.0863 (M-H)	342.3	C_15_H_18_O_9_	1-*O*-Caffeoylgalactose
6	6.7	195.0504 (M-H)	196.202	C_10_H_12_O_4_	2′-Hydroxy-4′,6′-dimethoxyacetophenone
7	7.72	431.0965 (M-H)	432.38	C_21_H_20_O_10_	Vitexin
8	9.31	206.0816 (M-Na)	229.279	C_14_H_15_NO_2_	*N*-[3-(4-Methoxyphenyl)propanoyl]pyrrole
9	13.9	209.0812 (M-H)	210.229	C_11_H_14_O_4_	Kadsuketanone A
10	14.75	239.0918 (M-H)	240.255	C_12_H_16_O_5_	3,4,5-Trimethoxydihydrocinnamic acid
11	16.71	237.0761 (M-H)	238.239	C_12_H_14_O_5_	2,4,5-Trimethoxycinnamic acid
12	18.3	225.1125 (M-H)	226.275	C_15_H_14_O_2_	1-Phenylethanol; benzoyl
13	19.6	361.1851 (M-H)	362.381	C_22_H_18_O_5_	Isochamanetin
14	23.46	229.1441 (M-H)	230.348	C_13_H_26_O_3_	Sarmentol A
15	25	243.1595 (M-H)	244.331	C_13_H_24_O_4_	Sarmentoic acid
16	25.74	279.1568 (M-Na)	302.238	C_15_H_10_O_7_	Quercetin
17	26.86	271.1906 (M-H)	272.256	C_15_H_12_O_5_	Naringenin
18	27.83	293.2112 (M-Na)	316.5	C_21_H_32_O_2_	1-(3,4-Methylenedioxyphenyl)-1*E*-tetradecene; (E)-form
19	29.63	265.1471 (M-H)	266.293	C_14_H_18_O_5_	3,4-Dihydroxy-5-(3-methyl-2-butenyl)benzoic acid; 3′-isomer, 2′-hydroxy, ethyl ester

HUVEC were isolated from human umbilical cords collected from the labor room of the Department of Obstetrics and Gynaecology, Hospital Kuala Lumpur. This study was approved by the Ethical Research Committee of Universiti Kebangsaan Malaysia Medical Centre and National Medical Research Registration (approval numbers: FF-2014-412 and NMRR-14-583-20729). Informed consent was obtained from all volunteers prior to delivery. HUVEC were isolated using collagenase infusion as described previously (Ugusman et al., 2010). The cells were cultured in endothelial cell medium (ECM) (ScienCell Research Laboratories, Inc., San Diego, CA) at 37°C in a humidified 5% CO_2_ incubator. The cells were not exposed to shear stress as the cell culture media did not fluctuate during the experiments and the cells were maintained in static conditions (Albuquerque et al., 2000). HUVEC were subcultured until they reached passage 3 at 80% confluency.

### 3-(4,5-Dimethylthiazol-2-Yl)-2,5-Diphenyltetrazolium Bromide (MTT) Assay for Determination of Cell Viability and Griess Assay for Determination of NO Level

Optimal dose of TNF-α was determined based on its effect on HUVEC viability and NO production. Briefly, 5 × 10^4^ HUVEC per well were seeded in a 96-well plate and allowed to attach for 24 h. The cells were then exposed to 10 to 40 ng/ml of TNF-α for 24 h. MTT assay was used to measure the viability of HUVEC while Griess assay was used to measure NO level in the culture medium. MTT assay was done according to a previous method (Ugusman et al., 2012). Briefly, 200 µl of MTT solution was added to each well, and the mixture was incubated for 4 h. Subsequently, the supernatants were aspirated, and the purple formazan crystal formed in each well was dissolved in 200 µl of DMSO. The absorbance of each well was read at 570 nm. A nitrate/nitrite colorimetric assay kit (Sigma-Aldrich, USA) was used to measure NO level indirectly in the cell culture medium as described previously (Ugusman et al., 2010). The principle of this assay is based on the measurement of total nitrite in the sample whereby nitrate reductase was utilized for enzymatic reduction of nitrate to nitrite. Total nitrite was measured at 540- nm absorbance after adding Griess reagents. Even though Griess reaction is less sensitive to quantitate NO generation compared to the direct method, it is the most extensively used method to measure NO indirectly; this method is fast and cheap and has a strong literature background (Csonka et al., 2015).

HUVEC were exposed to 50–300 µg/ml of AEPS for 24 h, and cell viability was measured using MTT assay to assess the extract’s cytotoxicity effect. In order to determine the optimal dose of AEPS for subsequent experiments, HUVEC were treated concomitantly with 30 ng/ml of TNF-α and three different concentrations of AEPS (150, 250, and 300 µg/ml) for 24 h. AEPS at these concentrations was used as it had shown potent antioxidant effects in a previous study (Hafizah et al., 2010). Subsequently, HUVEC viability and NO production were measured using MTT assay and Griess assay, respectively.

### Experimental Protocol

HUVEC at 1 × 10^5^ cell density were seeded onto six-well tissue culture plates and allowed to grow until it reached 80% confluency. The cells were then divided into four groups: control, treatment with 250 µg/ml of AEPS, treatment with 30 ng/ml of TNF-α, and concomitant treatment with 250 µg/ml of AEPS and 30 ng/ml of TNF-α for 24 h. The dose of TNF-α (30 ng/ml) was chosen as it was the first dose that significantly reduced HUVEC viability and NO production. As for AEPS, 250 µg/ml of AEPS was used as it significantly increased HUVEC viability and NO production when exposed to 30 ng/ml of TNF-α.

### Quantitative Real-Time Polymerase Chain Reaction (qRT-PCR) for Measurement of DDAH1 mRNA Expression

Extraction of RNA was done using TRI reagent (Molecular Research Center, Cincinnati, Ohio) as described previously (Ugusman et al., 2011). Following RNA extraction, cDNA synthesis was carried out using SuperScript^®^ III First-Strand Synthesis SuperMix for qRT-PCR kit (Invitrogen, Carlsbad, USA). Master mixes containing SYBR^®^ Select Master Mix (BIO-RAD Laboratories, Hercules, USA), cDNA samples, RNase- and DNase-free distilled water, and forward and reverse primers were prepared and pipetted into the PCR plate. Specific forward and reverse primers were used as follows: *DDAH1* (GenBank accession no. BC_033680), forward: 5′-ggacaaatcaacgaggtgct-3′, reverse: 5′-tagcggtggtcactcatctg-3′; and *GAPDH* (GenBank accession no. NM_002046), forward: 5′-tccctgagctgaacgggaag-3′, reverse: 5′-ggaggagtgggtgtcgctgt-3′. The reaction was conducted at initial denaturing at 95°C for 3 min; then involved 40 cycles of 61°C for 30 s, 95°C for 1 min, 55°C for 1 min, 70 cycles of 60°C for 10 s; and terminated by a cooling step at 4°C. Each experiment was performed in duplicate. The specificity of the PCRs was verified by analysis of melting curves and 1.5% agarose gel electrophoresis. The threshold cycle (C_T_) value was determined, and the relative mRNA expression of *DDAH* was analyzed as follows:

$$\text{Relative mRNA expression}=2^{-\Delta \Delta CT}$$

$$\Delta \Delta C_{T}=\;C_{T}GAPDH\;-\;C_{T}DDAH1$$

### ELISA for Measurement of DDAH1 Protein Level

DDAH1 protein level in HUVEC was measured using a human DDAH1 ELISA kit (Cloud-Clone Corp., USA) according to the manufacturer’s instructions. Briefly, 100 µl of HUVEC lysates was added into the plate and incubated for 2 h at 37°C. After incubation, liquid from each well was aspirated, and biotin-conjugated antibody specific to DDAH 1 was added. After washing, avidin conjugated to horseradish peroxidase (HRP) was added followed by 3,3′,5,5′-tetramethylbenzidine (TMB) substrate solution. Optical density (OD) of the wells was measured at 450 nm using a spectrophotometer.

### Colorimetric Assay for Measurement of DDAH Enzyme Activity

The activity of DDAH was determined based on a previous method (Prescott and Jones, 1969). The principle of this assay was based on the measurement of l-citrulline produced by DDAH from ADMA degradation in the sample in a timed reaction. Serially diluted l-citrulline standard of different concentrations from 0 to 100 mM was prepared. HUVEC lysates were centrifuged at 3,000 g for 10 min, and the supernatant was collected. Then, 250 µl of 20% sulfuric acid solution containing 0.5% w/v antipyrine and 250 µl of 5% acetic acid solution containing 0.8% w/v diacetyl monoxime were mixed. Subsequently, 100 µl of the mixture was added to each sample. The mixtures were incubated in a shaker incubator at 60°C for 110 min in a dark state before the absorbance was measured at 466 nm. Concentration of l-citrulline in the sample was calculated based on the standard curve. Total protein concentration in the samples was determined using Bradford assay (Bradford, 1976). DDAH enzyme activity is calculated based on the following formula:

DDAH activity=([L-citrulline]/110)/[total protein]

Total protein(mg)

### ELISA for Measurement of ADMA Concentration

ADMA concentration in HUVEC was determined using a human ADMA ELISA kit (Cloud-Clone Corp., USA) according to the manufacturer’s instructions. HUVEC lysate and biotin-labeled ADMA were added into their respective wells. The mixture was mixed and incubated for an hour at 37°C. After incubation, liquid was aspirated and washed. Then, avidin conjugated to HRP was added to each well and incubated for 30 min at 37°C. The processes of aspiration and washing were repeated. After the last wash, TMB substrate solution and sulfuric acid were added. OD of each well was measured at 450 nm using a spectrophotometer.

### Statistical Analysis

Data were analyzed using SPSS version 21.0 software. The Kolmogorov–Smirnov test was used to measure the normality of the data. Then, the data were analyzed using one-way ANOVA and *post hoc* Tukey test. The data were presented as mean ± standard error for the mean (SEM). Differences were considered significant at *P* < 0.05.

## Results

### AEPS Protected Against TNF-α-Induced Cytotoxicity

**Figure 2:**
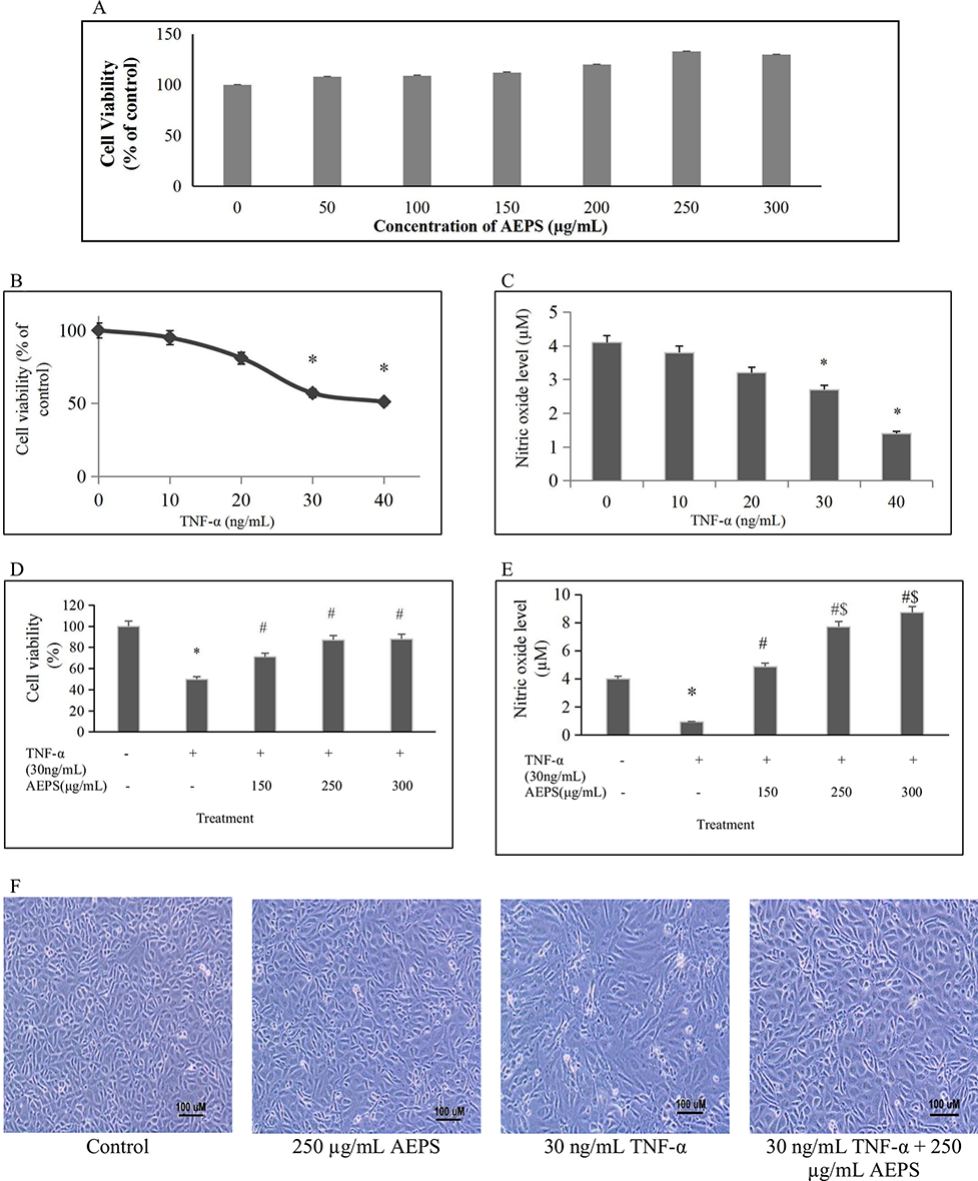
Protective effect of aqueous extract of *P. sarmentosum* leaves (AEPS) against tumor necrosis factor (TNF)-α-induced toxicity in human umbilical vein endothelial cells (HUVEC). Toxicity of AEPS in HUVEC was determined by 3-(4,5-dimethylthiazol-2-yl)-2,5-diphenyltetrazolium bromide (MTT) assay after treatment with 50–300 μg/ ml of AEPS for 24 h **(A)**. Cells were exposed to 10–40 ng/ml TNF-α for 24 h, cell viability was measured by MTT assay **(B)**, and NO level was measured by Griess assay **(C)**. In addition, HUVEC were treated with 30 ng/ml of TNF-α in combination with different concentrations of AEPS (150, 250, and 300 μg/ ml) for 24 h, and then cell viability **(D)** and NO level **(E)** were measured. Cell density and morphology were observed under microscope at 100× magnification **(F)**. All data are shown as the mean ± SEM of at least three independent experiments (*P < 0.05 compared with the control group, ^#^ *P* < 0.05 compared with the TNF-α group, ^$^ *P* < 0.05 compared with the group treated with 150 μg/ml of AEPS).

Exposure to varied concentrations of AEPS up to 300 μg/ml did not cause noticeable toxicity to HUVEC whose viability was 80% and above (**Figure 2A**). Treatment with different TNF-α concentrations ranging from 10 to 40 ng/ml induced cell death and reduced NO level in a dose-dependent manner with 30 and 40 ng/ml of TNF-α causing significant reduction in HUVEC viability (*P* < 0.05) and NO level (*P* < 0.05) (**Figures 2B, C**). Thus, 30 ng/ml of TNF-α as the first dose causing significant reduction in HUVEC viability and NO level was selected for subsequent experiments. In the presence of AEPS, TNF-α-induced cytotoxicity was significantly reduced, as determined by MTT (**Figure 2D**) and Griess (**Figure 2E**) assays, as well as cell density and morphological examination (**Figure 2F**). Treatment of TNF-α-induced HUVEC with 150, 250, and 300 μg/ml of AEPS also successfully increased HUVEC viability (*P* < 0.05) (**Figure 2D**). However, there was no significant difference in cell viability between these three doses of AEPS. In addition, Griess assay showed that treatment of TNF-α-induced HUVEC with 150, 250, and 300 μg/ml of AEPS increased NO production by HUVEC (*P* < 0.05) (**Figure 2E**). AEPS concentrations of 250 and 300 μg/ml stimulated more NO production compared to 150 μg/ml of AEPS (*P* < 0.05). However, there was no significant difference in the NO level between 250 and 300 μg/ml of AEPS. Based on the effects of AEPS on cell viability and NO production, 250 μg/ml of AEPS was selected as the dose for subsequent experiments. HUVEC exposed to 250 μg/ml of AEPS showed a normal cobblestone appearance and had a similar density as the control group (**Figure 2F**). TNF-α induced HUVEC had a striated appearance with less cell density compared to control. Treatment of TNF-α-induced HUVEC with AEPS improved the density, and the cells retained the cobblestone appearance.

### *DDAH1* mRNA Expression in HUVEC

**Figure 3:**
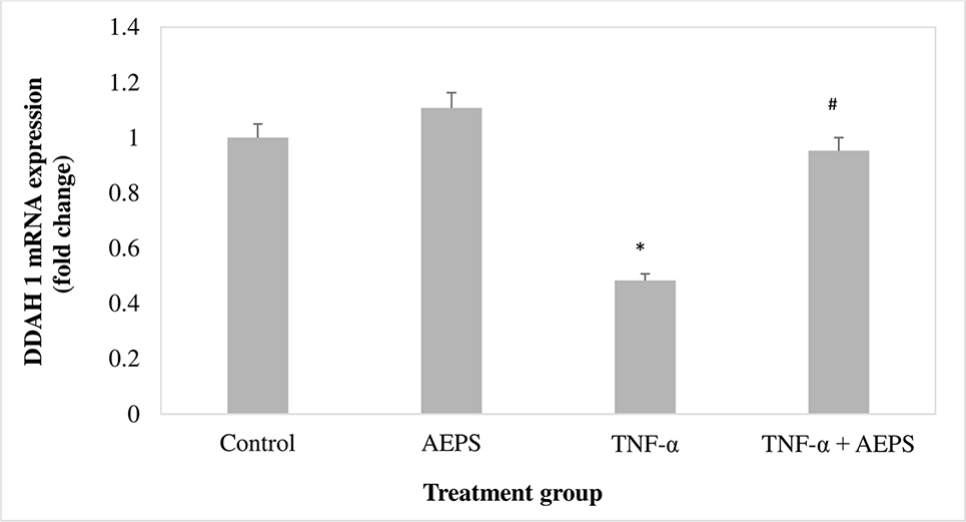
*DDAH1* messenger RNA (mRNA) expression in human umbilical vein endothelial cells (HUVEC). Data shown as mean ± SEM, *n* = 6 [*P < 0.05 compared with the control group, ^#^ *P* < 0.05 compared with tumor necrosis factor (TNF)-α group].

There was no significant difference in *DDAH1* mRNA expression for the AEPS group compared to the control group (*P* = 0.061) (**Figure 3**). TNF-α decreased *DDAH1* mRNA expression by 0.52-fold compared to control (*P* = 0.011). Treatment of TNF-α-induced HUVEC with AEPS increased *DDAH1* mRNA expression by 0.47-fold compared to the TNF-α group (*P* = 0.033).

### DDAH1 Protein Level in HUVEC

**Figure 4:**
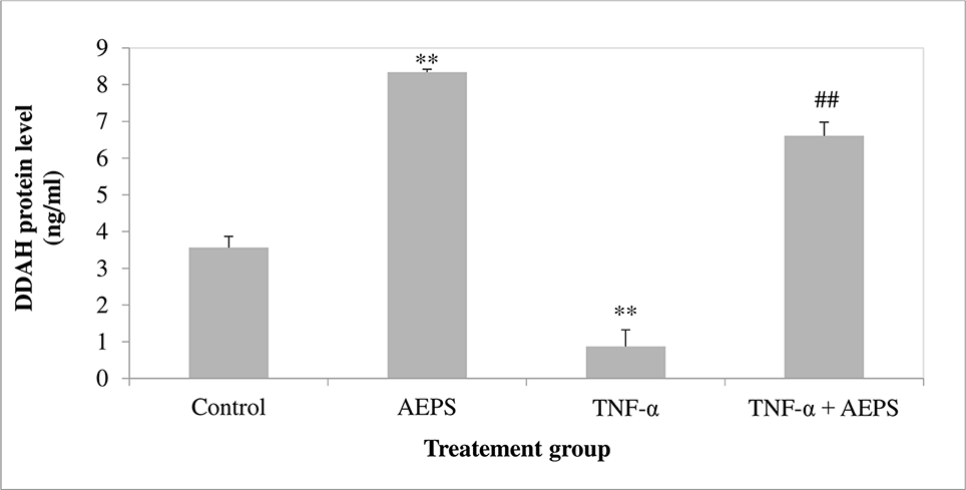
DDAH1 protein level in human umbilical vein endothelial cells (HUVEC). Data shown as mean ± SEM, *n* = 6 [**P < 0.01 compared with the control group, ^##^ *P* < 0.01 compared with the tumor necrosis factor (TNF)-α group].

AEPS increased DDAH1 protein level compared to control (*P* = 0.001) (**Figure 4**). TNF-α decreased DDAH1 protein level in HUVEC compared to control (*P* = 0.004). Treatment of TNF-α-induced HUVEC with AEPS increased DDAH1 protein level compared to the TNF-α group (*P* = 0.002).

### DDAH Enzyme Activity in HUVEC

**Figure 5:**
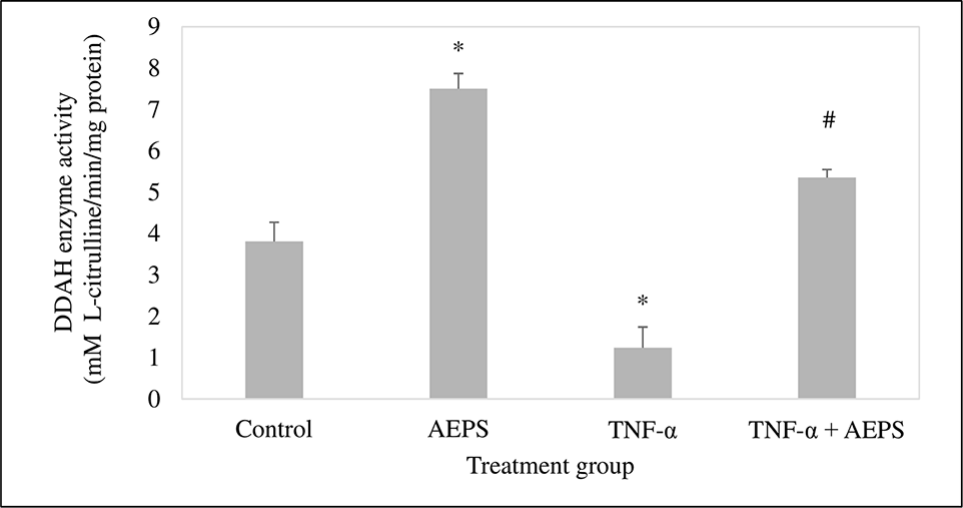
Dimethylarginine dimethylaminohydrolase (DDAH) enzyme activity in human umbilical vein endothelial cells (HUVEC). Data shown as mean ± SEM, *n* = 6 [*P < 0.05 compared with the control group, ^#^ *P* < 0.05 compared with the tumor necrosis factor (TNF)-α group].

AEPS induced DDAH activity compared with control (*P* = 0.023) (**Figure 5**). TNF-α caused a reduction in DDAH activity compared to control (*P* = 0.016). Treatment of TNF-α-induced HUVEC with AEPS increased DDAH activity compared to the TNF-α group (*P* = 0.039).

### ADMA Concentration in HUVEC

**Figure 6:**
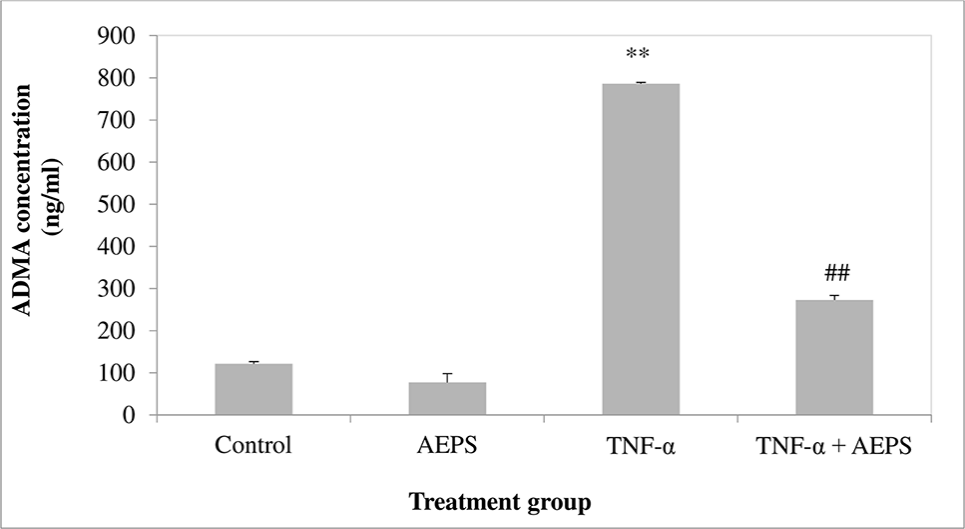
Asymmetric dimethylarginine (ADMA) concentration in human umbilical vein endothelial cells (HUVEC). Data shown as mean ± SEM, *n* = 6 [**P < 0.01 compared with THE control group, ^##^ *P* < 0.01 compared with the tumor necrosis factor (TNF)-α group].

There was no significant difference in ADMA level between the AEPS and control groups (*P* = 0.076) (**Figure 6**). Treatment of HUVEC with TNF-α resulted in an increase in ADMA compared to control (*P* = 0.0008). Treatment of TNF-α-induced HUVEC with AEPS successfully reduced ADMA concentration compared to the TNF-α group (*P* = 0.001).

### NO Production in HUVEC

**Figure 7:**
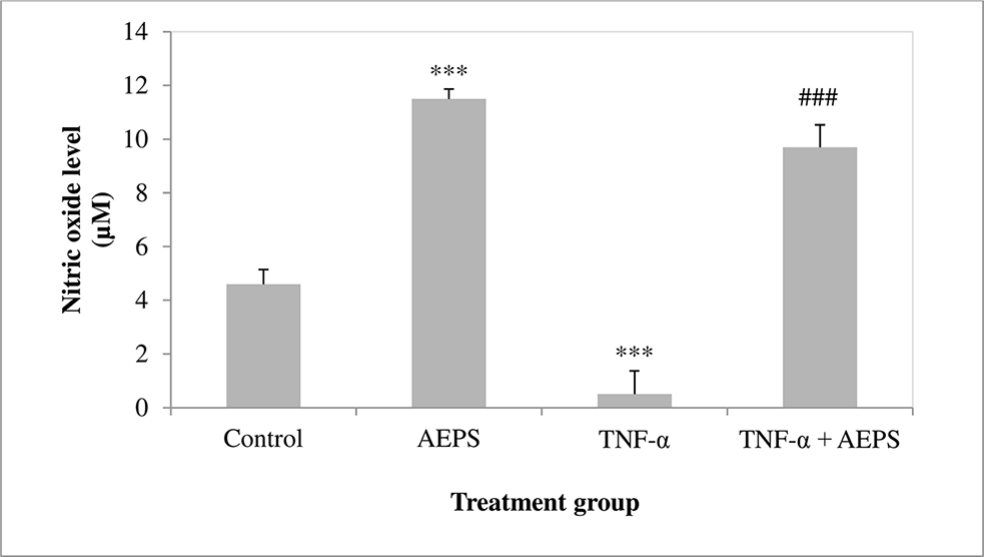
Nitric oxide level in human umbilical vein endothelial cells (HUVEC). Data shown as mean ± SEM, *n* = 6 [***P < 0.001 compared with the control group, ^###^ *P* < 0.001 compared with the tumor necrosis factor (TNF)-α group].

There was a significant increase in NO level produced by HUVEC treated with AEPS compared to the control group (*P* = 0.0001) (**Figure 7**). HUVEC induced by TNF-α showed a decrease in NO level compared to control (*P* = 0.0007). On the other hand, treatment of TNF-α-induced HUVEC with AEPS promoted NO production compared to the TNF-α group (*P* = 0.0001).

## Discussion

The main findings from this study were AEPS stimulated DDAH activity, thus decreasing ADMA level and increasing NO production in TNF-α-treated HUVEC. The results showed that TNF-α decreased *DDAH1* mRNA expression compared to control. This is in accordance with a previous study whereby mRNA expression of *DDAH1* is decreased in HUVEC treated with TNF-α or oxidized low-density lipoprotein (ox-LDL) (Ito et al., 1999). Induction with TNF-α also caused reduction in DDAH1 protein level and DDAH activity. This study was in conformity with the previous study whereby 250 U/mol of TNF-α reduced 60% of DDAH activity in endothelial cells (Vairappan et al., 2009). AEPS was able to increase *DDAH1* mRNA expression in HUVEC treated with TNF-α. AEPS increased *DDAH1* mRNA expression, causing more DDAH1 protein to be synthesized and DDAH activity to be increased. AEPS was reported to have anti-inflammatory action whereby it suppressed nuclear factor-κβ (NF-κβ) activation and cellular adhesion molecule expression in HUVEC treated with TNF-α (Ismail et al., 2018). Anti-TNF therapy resulted in reduced NF-κβ-driven inflammation and improved DDAH and eNOS expression in cirrhotic rats (Balasubramanian et al., 2017). Therefore, in this study, the anti-inflammatory activity of AEPS could be a factor contributing to improved DDAH activity in TNF-α-induced HUVEC.

Since DDAH plays an important role in degrading ADMA, reduction in DDAH activity leads to an increase in ADMA, which in turn reduces eNOS activity and NO production. High ADMA concentration in the plasma is a risk factor for development of atherosclerosis and cardiovascular diseases (Jawalekar et al., 2013). This study showed that treatment of HUVEC with TNF-α for 24 h caused an increase in ADMA level. A previous study showed that TNF-α-treated HUVEC had higher level of ADMA after 24 and 48 h of incubation (Eid et al., 2007). Another *in vitro* study reported that HUVEC exposed to TNF-α for 48 h showed eight times increase in ADMA level (Ito et al., 1999) and DDAH activity decreased to 60% of baseline value.

Based on previous studies, intracellular DDAH plays an important role in controlling ADMA concentrations (Vairappan et al., 2009). Endothelial cells exposed to *S*-2-amino-4(3-methylguanidino) butanoic acid had reduced DDAH activity and increased ADMA formation (MacAllister et al., 1996). Similarly, in another study, exposure of endothelial cells to TNF-α caused a reduction in DDAH activity that leads to accumulation of intracellular ADMA (Eid et al., 2007). Treatment of TNF-α-induced HUVEC with AEPS successfully decreased ADMA level. Since AEPS stimulates DDAH activity, more ADMA will be degraded to l-citrulline and dimethylamine, thus decreasing ADMA level. On the other hand, there are several therapeutic ways to reduce ADMA. Previous studies have reported that l-arginine supplementation enhances endothelial function in hypercholesterolemic rabbits by reducing ADMA (Brinkmann et al., 2015). A clinical study on patients with rheumatoid arthritis showed a significant reduction in ADMA after 3 months of treatment with TNF-α inhibitors by increasing endothelial progenitor cells (Spinelli et al., 2013).

Endothelial NO is mainly synthesized by eNOS from l-arginine. Treatment of HUVEC with TNF-α reduced NO production. By inhibiting DDAH activity, TNF-α reduces ADMA degradation. ADMA inhibits eNOS competitively, thus reducing NO synthesis (Czarnecka et al., 2017). Furthermore, TNF-α reduces eNOS mRNA expression and the stability of eNOS mRNA, resulting in further reduction of NO production in HUVEC (Guijun et al., 2008). Treatment of TNF-α-induced HUVEC with AEPS promoted NO production. Since AEPS was able to stimulate DDAH activity, less ADMA was available to inhibit eNOS. This resulted in the increase in NO level. The results were in accordance with previous study whereby AEPS leaves stimulated eNOS activity and NO production in HUVEC (Ugusman et al., 2010).

The results also showed that AEPS promoted NO generation in both the absence and presence of TNF-α. In an earlier study, AEPS leaves had been shown to stimulate NO production in the absence and presence of hydrogen peroxide (H_2_O_2_) (Ugusman et al., 2010). Even in baseline culture condition without H_2_O_2_ treatment, AEPS alone was able to increase eNOS mRNA expression, eNOS protein level, and eNOS activity in HUVEC. This resulted in increased NO production by the cells (Ugusman et al., 2010). In this study, at baseline condition, AEPS alone promoted NO generation together with increased DDAH protein level and activity. Increased DDAH activity caused ADMA level to be maintained at a low baseline level, hence promoting eNOS activity and NO production. This result suggested that AEPS improved endothelial function by augmenting endothelial NO production in both healthy and diseased states.

Antioxidants are known to enhance NO by scavenging reactive oxygen species (ROS), hence protecting NO against oxidative destruction by ROS (Ignarro et al., 2006). As AEPS has an antioxidant effect, there may be a possibility that the increase in NO in this study is contributed by its ROS scavenging action too. However, this factor is beyond the scope of our study. It will be interesting to look at this factor in future experiments.

LC–MS Orbitrap full-scan analysis of AEPS leaves used in this study showed the presence of flavonoids such as naringenin, quercetin, and vitexin. Naringenin had been shown to stimulate eNOS and NO in endothelial cells exposed to high glucose (Qin et al., 2016). Quercetin caused an increase in plasma NO and eNOS activity in vascular tissues of hypertensive rats (Yamamoto and Oue, 2006). Besides, quercetin increased DDAH activity and decreased ADMA concentration in rat’s liver (Trocha et al., 2012). Consumption of biscuits containing bioactive complex of quercetin, selenium, catechins, and curcumin reduced ADMA level in the plasma of healthy men (Madaric et al., 2013). Quercetin also inhibited ADMA-induced apoptosis in glomerular endothelial cells (Guo et al., 2014).

However, to date, there is no study done on the effect of vitexin on the DDAH–ADMA–NO pathway yet. Nevertheless, vitexin was proven to promote cell viability, suppressed apoptosis and reduced oxidative stress in HUVEC treated with ox-LDL (Zhang et al., 2017). Besides, vitexin inhibited overexpression of pro-inflammatory cytokines such as IL-1β, IL-6, TNF-α, and cellular adhesion molecules in ox-LDL-induced HUVEC by activating 5′-adenosine monophosphate-activated protein kinase (AMPK) signaling (Zhang et al., 2017). In lipopolysaccharide (LPS)-activated RAW 264.7 cells, vitexin was able to reduce pro-inflammatory mediators by inhibiting the expression of transcriptional factors such as p-p38, p-ERK1/2, and p-JNK (Rosa et al., 2016). Since anti-TNF therapy resulted in reduced inflammation and improved DDAH expression in rats (Balasubramanian et al., 2017), the anti-inflammatory activity of vitexin could also be a contributing factor to the effects of AEPS on DDAH activity in this study.

Previous phytochemical analysis showed that *P. sarmentosum* contains other active compounds such as rutin, vitamin E, carotenes, tannins, and xanthophyll (Miean and Mohamed, 2001). An *in vivo* study on ischemia/reperfusion rat models reported that rutin stimulated the DDAH/NOS pathway by upregulating both eNOS and *DDAH1* expression (Lanteri et al., 2007). Besides, high-dose vitamin E supplementation potentially reduced ADMA level in patients with chronic renal insufficiency by improving DDAH activity and NO bioavailability (Jiang et al., 2002). In a Japanese population, high serum carotenoid levels such as α-carotene and β-carotene were linked to low ADMA level (Watarai et al., 2014). As this study used crude extract of *P. sarmentosum* and not its purified active compound, this study was unable to identify the specific component of the extract that mediated the observed effects. However, it is suggested that the effects were likely due to the active compounds mentioned above.

## Study Limitations

This study did not evaluate the detailed intracellular signaling pathway by which AEPS acts on the endothelial cells. Besides, the present study did not consider the possibility of the results to be related to the reduction of oxidative stress by the antioxidant effect of AEPS. Further studies incorporating endothelial response to shear stress as well as functional studies should be performed in order to extrapolate the findings from this *in vitro* study to what is observed physiologically.

## Conclusion

The present study shows that AEPS promotes endothelial NO production by stimulating DDAH activity and thus decreases ADMA level in TNF-α-induced HUVEC. Based on the vasoprotective and anti-atherosclerotic effects of endothelial NO, AEPS has the potential to reduce the risk of endothelial dysfunction and atherosclerosis. However, further studies using isolated bioactive components from AEPS are required in order to support the therapeutic potential of AEPS for endothelial dysfunction and atherosclerosis.

## Data Availability

All datasets generated for this study are included in the manuscript.

## Ethics Statement

This study was approved by the Ethical Research Committee of Universiti Kebangsaan Malaysia Medical Centre and National Medical Research Registration (approval numbers: FF-2014-412 and NMRR-14-583-20729). Informed written consent was obtained from all participants.

## Author Contributions

AU and CH conceptualized the study and designed the experiment. US and JL performed the experiments and analyzed the data. AU, CH, AA, JL, and US analyzed and interpreted the data. AU, CH, AA, JL, and US wrote the manuscript and performed critical analysis and the necessary revisions of the work. All authors read and approved the final manuscript.

## Funding

This study was supported by research grants from the Ministry of Higher Education, Malaysia (Grant No.: FRGS/1/2014/SKK03/UKM/02/1), and Universiti Kebangsaan Malaysia (Grant No: GGPM-2013-029). The funders had no role in study design, data collection and analysis, decision to publish, or preparation of the manuscript.

## Conflict of Interest Statement

The authors declare that the research was conducted in the absence of any commercial or financial relationships that could be construed as a potential conflict of interest.

## References

[B1] Adel A. Zaiton Z. Faizah O. Srijit D. Santhana R. Nor-Anita N. (2010). Aqueous extract of *Piper sarmentosum* decreases atherosclerotic lesions in high cholesterolemic experimental rabbits. Lipids Health Dis. 9, 44. 10.1186/1476-511X-9-44 20433693 PMC2877048

[B2] Albuquerque M. L. C. Waters C. M. Savla U. Schnaper H. W. Flozak A. S. (2000). Shear stress enhances human endothelial cell wound closure *in vitro*. Am. J. Physiol. Heart Circ. Physiol. 279 (1), H293–H302. 10.1152/ajpheart.2000.279.1.H293 10899069

[B3] Balasubramanian V. Mehta G. Jones H. Sharma V. Davies N. A. Jalan R. (2017). Post-transcriptional regulation of hepatic DDAH1 with TNF blockade leads to improved eNOS function and reduced portal pressure in cirrhotic rats. Sci. Rep. 7 (1), 179000. 10.1038/s41598-017-18094-3 PMC5738445 29263339

[B4] Benjamin E. J. Blaha M. J. Chiuve S. E. Cushman M. Das S. R. Deo R. (2017). American Heart Association Statistics Committee and Stroke Statistics Subcommittee. Heart disease and stroke statistics—2017 update: a report from the American Heart Association. Circulation 135 (10), e146–e603. 10.1161/CIR.0000000000000485 28122885 PMC5408160

[B5] Bradford M. M. (1976). A rapid and sensitive method for the quantitation of microgram quantities of protein utilizing the principle of protein-dye binding. Anal. Biochem. 72, 248–254. 10.1016/0003-2697(76)90527-3 942051

[B6] Brinkmann S. J. H. Worner E. A. Buijs N. Richir M. Cynober L. van Leeuwen P. A. (2015). The arginine/ADMA ratio is related to the prevention of atherosclerotic plaques in hypercholesterolemic rabbits when giving a combined therapy with atorvastatine and arginine. Int. J. Mol. Sci. 16 (6), 12230–12242. 10.3390/ijms160612230 26035753 PMC4490441

[B7] Bulau P. Zakrzewicz D. Kitowska K. Leiper J. Gunther A. Grimminger F. (2007). Analysis of methylarginine metabolism in the cardiovascular system identifies the lung as a major source of ADMA. Am. J. Physiol. Lung Cell Mol. Physiol. 292, L18–L24. 10.1152/ajplung.00076.2006 16891395

[B8] Chan E. W. C. Wong S. K. (2014). Phytochemistry and pharmacology of three *Piper* species: an update. IJP. 1 (9), 534–544. 10.13040/IJPSR.0975-8232.IJP

[B9] Chaveerach A. Mokkamul P. Sudmoon R. Tanee T. (2008). Ethnobotany of the genus *Piper* (Piperaceae) in Thailand. Ethnobot. Res. Appl. 4, 223–231. 10.17348/era.4.0.223-231

[B10] Csonka C. Páli T. Bencsik P. Görbe A. Ferdinandy P. Csont T. (2015). Measurement of NO in biological samples. Br. J. Pharmacol. 172 (6), 1620–1632. 10.1111/bph.12832 24990201 PMC4369268

[B11] Czarnecka A. Milewski K. Zielińska M. (2017). Asymmetric dimethylarginine and hepatic encephalopathy: cause, effect or association? Neurochem. Res. 42 (3), 750–761. 10.1007/s11064-016-2111-x 27885576 PMC5357500

[B12] Eid H. M. A. Lyberg T. Arnesen H. Seljeflot I. (2007). Insulin and adiponectin inhibit the TNF-α induced ADMA accumulation in human umbilical endothelial cells: the role of DDAH. Atherosclerosis 194 (2), 1–8. 10.1016/j.atherosclerosis.2006.11.008 17141242

[B13] Guijun Y. You B. Chen S. P. Liao J. K. Sun J. (2008). Tumor necrosis factor-α downregulates endothelial nitric oxide synthase mRNA stability *via* translation elongation factor 1-α1. Circ. Res. 103, 591–597. 10.1161/CIRCRESAHA.108.173963 18688046 PMC2753820

[B14] Guo W. Ding J. Zhang A. Dai W. Liu S. Diao Z. (2014). The inhibitory effect of quercetin on asymmetric dimethylarginine-induced apoptosis is mediated by the endoplasmic reticulum stress pathway in glomerular endothelial cells. Int. J. Mol. Sci. 15 (1), 484–503. 10.3390/ijms15010484 24451129 PMC3907821

[B15] Hafizah A. H. Zaiton Z. Zulkhairi A. Mohd Ilham A. Nor Anita M. M. Zaleha A. M. (2010). *Piper sarmentosum* as an antioxidant on oxidative stress in human umbilical vein endothelial cells induced by hydrogen peroxide. J. Zhejiang Univ. Sci. B. 11, 357–365. 10.1631/jzus.B0900397 20443214 PMC2865838

[B16] Ignarro L. J. Byrns R. E. Sumi D. de Nigris F. Napoli C. (2006). Pomegranate juice protects nitric oxide against oxidative destruction and enhances the biological actions of nitric oxide. Nitric Oxide 15 (2), 93–102. 10.1016/j.niox.2006.03.001 16626982

[B17] Ishizaka M. Nagai K. Iwanaga M. Imamura M. Azuma H. (2007). Possible involvement of enhanced arginase activity due to up-regulated arginases and decreased hydroxyarginine in accelerating intimal hyperplasia with hyperglycemia. Vascul. Pharmacol. 47, 272–280. 10.1016/j.vph.2007.08.001 17804300

[B18] Ismail S. M. Sundar U. M. Ugusman A. Hui C. K. Aminuddin A. (2018). *Piper sarmentosum* attenuates TNF-α-induced VCAM-1 and ICAM-1 expression in human umbilical vein endothelial cells. J. Taibah. Univ. Med. Sci. 13 (3), 225–231. 10.1016/j.jtumed.2018.01.003 31435328 PMC6694970

[B19] Ito A. Tsao P. S. Adimoolam S. Kimoto M. Ogawa T. Cooke J. P. (1999). Novel mechanism for endothelial dysfunction: dysregulation of dimethylarginine dimethylaminohydrolase. Circulation 99, 3092–3095. 10.1161/01.CIR.99.24.3092 10377069

[B20] Jawalekar S. L. Karnik A. Bhutey A. (2013). Risk of cardiovascular diseases in diabetes mellitus and serum concentration of asymmetrical dimethylarginine. Biochem. Res. Int. 1–6. 10.1155/2013/189430 PMC3804277 24187621

[B21] Jiang L. Li N. S. Li Y. J. Deng H. W. (2002). Probucol preserves endothelial function by reduction of the endogenous nitric oxide synthase inhibitor level. Br. J. Pharmacol. 135, 1175–1182. 10.1038/sj.bjp.0704563 11877324 PMC1573227

[B22] Lanteri R. Acquaviva R. DiGiacomo C. Sorrenti V. Destri G.L. Santangelo M. (2007). Rutin in rat liver ischemia/reperfusion injury: effect on DDAH/NOS pathway. Microsurgery 27 (4), 245–251. 10.1002/micr.20345 17477412

[B23] Liu X. Hou L. Xu D. Chen A. Yang L. Zhuang Y. (2016). Effect of asymmetric dimethylarginine (ADMA) on heart failure development. Nitric Oxide 54, 73–81. 10.1016/j.niox.2016.02.006 26923818 PMC5017793

[B24] MacAllister R. J. Parry H. Kimoto M. Ogawa T. Russel R. J. Hodson H. (1996). Regulation of nitric oxide synthesis by dimethylarginine dimethylaminohydrolase. Br. J. Pharmacol. 119, 1533–1540. 10.1111/j.1476-5381.1996.tb16069.x 8982498 PMC1915783

[B25] Madaric A. Kadrabova J. Krajcovicova-Kudlackova M. Valachovicova M. Spustova V. Mislanova C. (2013). The effect of bioactive complex of quercetin, selenium, catechins and curcumin on cardiovascular risk markers in healthy population after a two month consumption. Bratisl. Lek. Listy. 114 (2), 84–87. 10.4149/BLL_2013_019 23331204

[B26] Miean K. H. Mohamed S. (2001). Flavonoid (myricetin, quercetin, kaempferol, luteolin, and apigenin) content of edible tropical plants. J. Agric. Food Chem. 49, 3106–3112. 10.1021/jf000892m 11410016

[B27] Pope A. J. Karuppiah K. Cardounel A. J. (2009). Role of the PRMT–DDAH–ADMA axis in the regulation of endothelial nitric oxide production. Pharmacol. Res. 60, 461–465. 10.1016/j.phrs.2009.07.016 19682581 PMC2767407

[B28] Prescott L. M. Jones M. E. (1969). Modified methods for the determination of carbamyl aspartate. Anal. Biochem. 32, 408–419. 10.1016/S0003-2697(69)80008-4 5361395

[B29] Qin W. Ren B. Wang S. Liang S. He B. Shi X. (2016). Apigenin and naringenin ameliorate PKCβII-associated endothelial dysfunction *via* regulating ROS/caspase-3 and NO pathway in endothelial cells exposed to high glucose. Vascul. Pharmacol. 85, 39–49. 10.1016/j.vph.2016.07.006 27473516

[B30] Rosa S. I. G. Rios-Santos F. Balogun S. O. de Oliveira Martins D. T. (2016). Vitexin reduces neutrophil migration to inflammatory focus by down-regulating pro-inflammatory mediators *via* inhibition of p38, ERK1/2 and JNK pathway. Phytomedicine. 23 (1), 9–17. 10.1016/j.phymed.2015.11.003 26902402

[B31] Spinelli F. R. Metere A. Barbati C. Pierdominici M. Iannuccelli C. Lucchino B. (2013). Effect of therapeutic inhibition of TNF on circulating endothelial progenitor cells in patients with rheumatoid arthritis. Mediators Inflamm. 1–8. 10.1155/2013/537539 PMC3810060 24222719

[B32] Su J. B. (2015). Vascular endothelial dysfunction and pharmacological treatment. World J. Cardiol. 7 (11), 719–741. 10.4330/wjc.v7.i11.719 26635921 PMC4660468

[B33] Sukhovershin R. A. Yepuri G. Ghebremariam Y. T. (2015). Endothelium-derived nitric oxide as an antiatherogenic mechanism: implications for therapy. Methodist Debakey Cardiovasc. J. 11 (3), 166–171. 10.14797/mdcj-11-3-166 26634024 PMC4666423

[B34] Trocha M. Merwid-Lad A. Szuba A. Sozanski T. Magdalan J. Szelag A. (2012). Effect of quercetin-5′-sulfonic acid sodium salt on SOD activity and ADMA/DDAH pathway in extracorporeal liver perfusion in rats. Adv. Clin. Exp. Med. 21 (4), 423–431. 23240447

[B35] Ugusman A. Zakaria Z. Hui C. K. Nordin N. (2011). *Piper sarmentosum* inhibits ICAM-1 and Nox4 gene expression in oxidative stress-induced human umbilical vein endothelial cells. BMC Complement. Altern. Med. 11, 31. 10.1186/1472-6882-11-31 21496279 PMC3090383

[B36] Ugusman A. Zakaria Z. Hui C. K. Nordin N. A. M. M. Mahdy Z. A. (2012). Flavonoids of Piper sarmentosum and its cytoprotective effects against oxidative stress. EXCLI J. 11, 705-714. 10.17877/DE290R-10356 27847456 PMC5099915

[B37] Ugusman A. Zakaria Z. Hui C. K. Nordin N. (2010). *Piper sarmentosum* increases nitric oxide production in oxidative stress: a study on human umbilical vein endothelial cells. Clinics 65, 709–714. 10.1590/S1807-59322010000700010 20668629 PMC2910860

[B38] Vairappan B. (2015). Endothelial dysfunction in cirrhosis: role of inflammation and oxidative stress. World J. Hepatol. 7 (3), 443–459. 10.4254/wjh.v7.i3.443 25848469 PMC4381168

[B39] Vairappan B. Sharma V. Winstanley A. Davies N. Shah N. Jalan R. (2009). Modulation of the DDAH–ADMA pathway with the Farnesoid receptor (FXR) agonist INT-747 restores hepatic eNOS activity and lowers portal pressure in cirrhotic rats. Hepatology 50, 336A–337A.

[B40] Watarai R. Suzuki K. Ichino N. Osakabe K. Sugimoto K. Yamada H. (2014). Association between serum levels of carotenoids and serum asymmetric dimethylarginine levels in Japanese subjects. J. Epidemiol. 24 (3), 250–257. 10.2188/jea.JE20130137 24727752 PMC4000773

[B41] Yamamoto Y. Oue E. (2006). Antihypertensive effect of quercetin in rats fed with a high-fat high-sucrose diet. Biosci. Biotechnol. Biochem. 70 (4), 933–939. 10.1271/bbb.70.933 16636461

[B42] Zainudin M. M. Zakaria Z. Nordin N. A. M. M. (2015). The use of *Piper sarmentosum* leaves aqueous extract (Kadukmy™) as antihypertensive agent in spontaneous hypertensive rats. BMC Complement. Altern. Med. 15, 54. 10.1186/s12906-015-0565-z 25887182 PMC4367816

[B43] Zainudin M. M. Zakaria Z. Nordin N. A. M. M. Othman F. (2013). Does oral ingestion of *Piper sarmentosum* cause toxicity in experimental animals? Evid. Based. Complement. Alternat. Med. 1–9. 10.1155/2013/705950 PMC3817794 24228062

[B44] Zhang P. Hu X. Xu X. Chen Y. Bache R.J. (2011). Dimethylarginine dimethylaminohydrolase 1 modulates endothelial cell growth through NO and Akt. Arterioscler. Thromb. Vasc. Biol. 31 (4), 890–897. 10.1161/ATVBAHA.110.215640 21212404 PMC3064458

[B45] Zhang S. Guo C. Chen Z. Zhang P. Li J. Li Y. (2017). Vitexin alleviates ox-LDL-mediated endothelial injury by inducing autophagy *via* AMPK signaling activation. Mol. Immunol. 85, 214–221. 10.1016/j.molimm.2017.02.020 28288411

